# A Unified Hierarchical Multiscale Fusion Framework for Drug–Target Affinity Prediction: From Benchmark Performance to Nanomolar Inhibitor Discovery

**DOI:** 10.1002/advs.77345

**Published:** 2026-08-21

**Authors:** Shuo Liu, Xiang Zhang, Haixia Feng, Yuquan Li, Xiaoqing Gong, Yong Liang, Xiaojun Yao, Huanxiang Liu

**Affiliations:** ^1^ Chinese Medicine Guangdong Laboratory (Hengqin Laboratory) Guangdong‐Macao In‐Depth Cooperation Zone in Hengqin Guangdong China; ^2^ The Second Affiliated Hospital of Guangzhou University of Chinese Medicine Guangzhou China; ^3^ State Key Laboratory of Public Big Data College of Computer Science and Technology Guizhou University Guiyang China; ^4^ Faculty of Applied Sciences Macao Polytechnic University Macao China

**Keywords:** contrastive learning, drug–target affinity prediction, HPK1 inhibitors, lead identification, multimodal fusion

## Abstract

Accurately predicting drug–target affinity (DTA) is crucial for accelerating virtual screening and guiding lead optimization in drug discovery. However, current computational approaches face a critical trade‐off: interaction‐free models lack fine‐grained binding details, while interaction‐based models overlook higher‐order contextual and functional patterns. This limitation hinders both prediction performance and real‐world generalization. To overcome this, we propose MF‐Net, a unified hierarchical multiscale fusion framework that integrates sequence‐, atomic‐, and fragment‐level representations to model drug–target interactions across complementary scales. MF‐Net achieves state‐of‐the‐art performance on the PDBBind v2016 benchmark and demonstrates strong early enrichment across multiple virtual screening datasets. Additionally, ADP‐Glo assays confirm that the MF‐Net‐guided virtual screening pipeline identifies seven novel nanomolar inhibitors targeting hematopoietic progenitor kinase 1 (HPK1). Among them, one compound achieves sub‐nanomolar activity (IC_50_ = 0.41 nM), outperforming the positive control inhibitor Sunitinib. These results demonstrate that MF‐Net not only excels on standard benchmarks but also delivers tangible lead discovery outcomes, underscoring its practical value for structure‐based drug design.

## Graphical abstract

A multimodal fusion framework integrating sequence, atomic, and fragment representations captures drug–target interactions across multiple scales. The model delivers strong predictive performance and enables efficient virtual screening. Applied to hematopoietic progenitor kinase 1 (HPK1), it identifies structurally diverse inhibitors with nanomolar potency, demonstrating a direct path from computational prediction to experimental lead discovery. This work provides a powerful, generalizable tool for translating AI‐driven screening into tangible drug development.

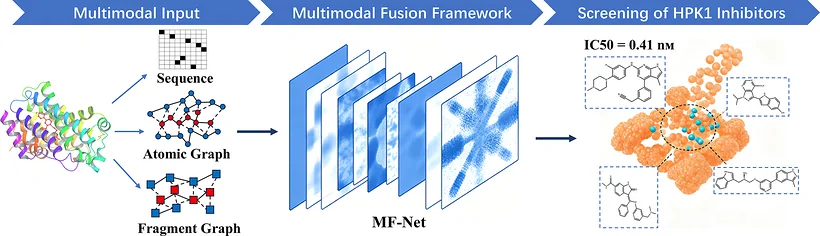

## 1 Introduction

Drug discovery remains a costly and time‐intensive process, largely due to the difficulty of identifying truly bioactive compounds from vast chemical space [1, 2]. While advances in high‐throughput screening have improved efficiency, the ability to reliably prioritize promising candidates before experimental validation remains a central bottleneck [3, 4, 5, 6, 7, 8, 9, 10]. Computational prediction of drug–target affinity (DTA), which estimates the binding strength between small molecules and protein targets [11, 12, 13], has therefore emerged as a critical component in modern drug discovery pipelines.

Despite substantial progress, existing computational approaches still struggle to translate predictive performance into practical discovery outcomes. Traditional physics‐based methods, such as molecular dynamics and quantum mechanical simulations, provide detailed and accurate descriptions of molecular interactions but are prohibitively expensive for large‐scale screening [14]. Molecular docking offers a more efficient alternative, yet often sacrifices accuracy due to simplified scoring functions [15]. More recently, deep learning‐based methods have emerged as powerful alternatives, leveraging large‐scale data to learn complex patterns of biomolecular interactions [16]. However, current models typically adopt either interaction‐free representations, which capture global sequence or structural context but lack explicit modeling of binding interfaces [17, 18, 19, 20, 21, 22], or interaction‐based representations, which focus on atomistic interactions but often overlook higher‐level functional and contextual information [23, 24, 25, 26, 27, 28]. Moreover, existing multimodal fusion approaches typically aggregate representations from different molecular entities (e.g., protein, ligand, and complex) as parallel inputs [5, 29, 30, 31], lacking an explicit hierarchical interaction structure across molecular scales.

However, binding affinity is not determined at a single level of representation but emerges from multimodal information according to different levels of drug–target interaction, including global context, fine‐grained atomic interactions, and higher‐level functional substructures. Specifically, sequence‐level features encode global physicochemical context and evolutionary constraints; atomic‐level interactions describe fine‐grained spatial geometry; and fragment‐level representations capture functional substructures that mediate specific recognition events. These complementary factors are particularly critical for challenging targets such as kinases. Hematopoietic progenitor kinase 1 (HPK1), a key negative regulator of immune signaling, has recently emerged as a promising target for cancer immunotherapy [32]. However, the identification of potent and selective HPK1 inhibitors remains challenging due to the highly conserved nature of kinase domains and the need to simultaneously optimize atomic interactions and functional motif recognition.

Motivated by this challenge, we reformulate DTA prediction as a hierarchical multiscale representation learning problem and propose MF‐Net, which models drug–target interactions across sequence, atomic, and fragment levels within a unified framework to capture complementary multiscale information. This hierarchical design enables MF‐Net to bridge global biochemical context, local binding geometry, and functional recognition patterns, rather than merely aggregating independent entity representations. Furthermore, a contrastive alignment strategy is introduced to enforce cross‐modal consistency, improving robustness to heterogeneous data and enhancing the integration of diverse molecular features. By jointly modeling global context, local geometry, and functional substructures, MF‐Net enables a more comprehensive and coherent representation of molecular recognition. Extensive experiments demonstrate that MF‐Net achieves state‐of‐the‐art performance on the PDBBind v2016 benchmark and multiple structure‐based virtual screening datasets, highlighting its strong generalization ability. More importantly, its practical utility is validated in a prospective virtual screening campaign targeting HPK1. This screening capability is further supported by ADP‐Glo assays, where seven of twenty compounds selected through the MF‐Net‐guided virtual screening workflow exhibited inhibitory activity against HPK1, with IC_50_ values of 5.63 nM (HQ2026‐01), 11.24 nM (HQ2026‐02), 0.41 nM (HQ2026‐03), 431.20 nM (HQ2026‐04), 105.70 nM (HQ2026‐05), 26.26 nM (HQ2026‐06), 5697.00 nM (HQ2026‐07), respectively. These hits display diverse chemical scaffolds, underscoring the model's ability to identify structurally novel and biologically relevant inhibitors. Together, these results demonstrate that MF‐Net not only advances the predictive accuracy of DTA models but also effectively bridges computational modeling and experimental validation, providing a robust and scalable framework for real‐world drug discovery.

## 2 Results and Discussion

### 2.1 The Overall Architecture of MF‐Net Framework

Figure 1 illustrates the overall framework of the proposed MF‐Net, which unifies interaction‐free and interaction‐based strategies through a multimodal fusion strategy for comprehensive representation of drug–target interactions. The framework is designed to capture global, local, and functional information across multiple molecular scales, and it consists of four core modules: the sequence feature extraction module, the atomic feature extraction module, the fragment feature extraction module, and the fusion and prediction module. First, the sequence feature extraction module adopts an interaction‐free encoding strategy, where protein sequences and ligand features are independently encoded to generate global contextual representations. This module captures overall structural and physicochemical patterns that reflect the global binding tendency without requiring explicit atomic interactions. Second, the atomic feature extraction module represents the drug–target binding pocket as atomic graphs. It employs SchNet to learn fine‐grained atomic embeddings and refines these through an intermolecular graph convolution network, enabling accurate modeling of atomic‐scale physical interactions. Third, the fragment feature extraction module adopts a similar interaction‐based strategy but focuses on higher‐level functional representations. It constructs fragment‐level graphs for protein residues and ligand motifs and captures their functional interactions through intermolecular graph convolution. Finally, the fusion and prediction module integrates the representations from the three levels (sequence, atomic, and fragment) using a convolutional neural network (CNN), which adaptively learns the relative importance of each modality. The fused representation is then fed into a multilayer perceptron (MLP) to predict DTA. To enhance cross‐modal consistency, MF‐Net introduces a contrastive alignment loss based on feature similarity, encouraging the model to learn complementary and consistent information across modalities. In addition, we incorporate the E‐DIS framework [19] to capture domain‐generic and domain‐specific features. Through the synergistic operation of these modules, MF‐Net achieves a unified and interpretable multiscale understanding of drug–target interactions.

**FIGURE 1:**
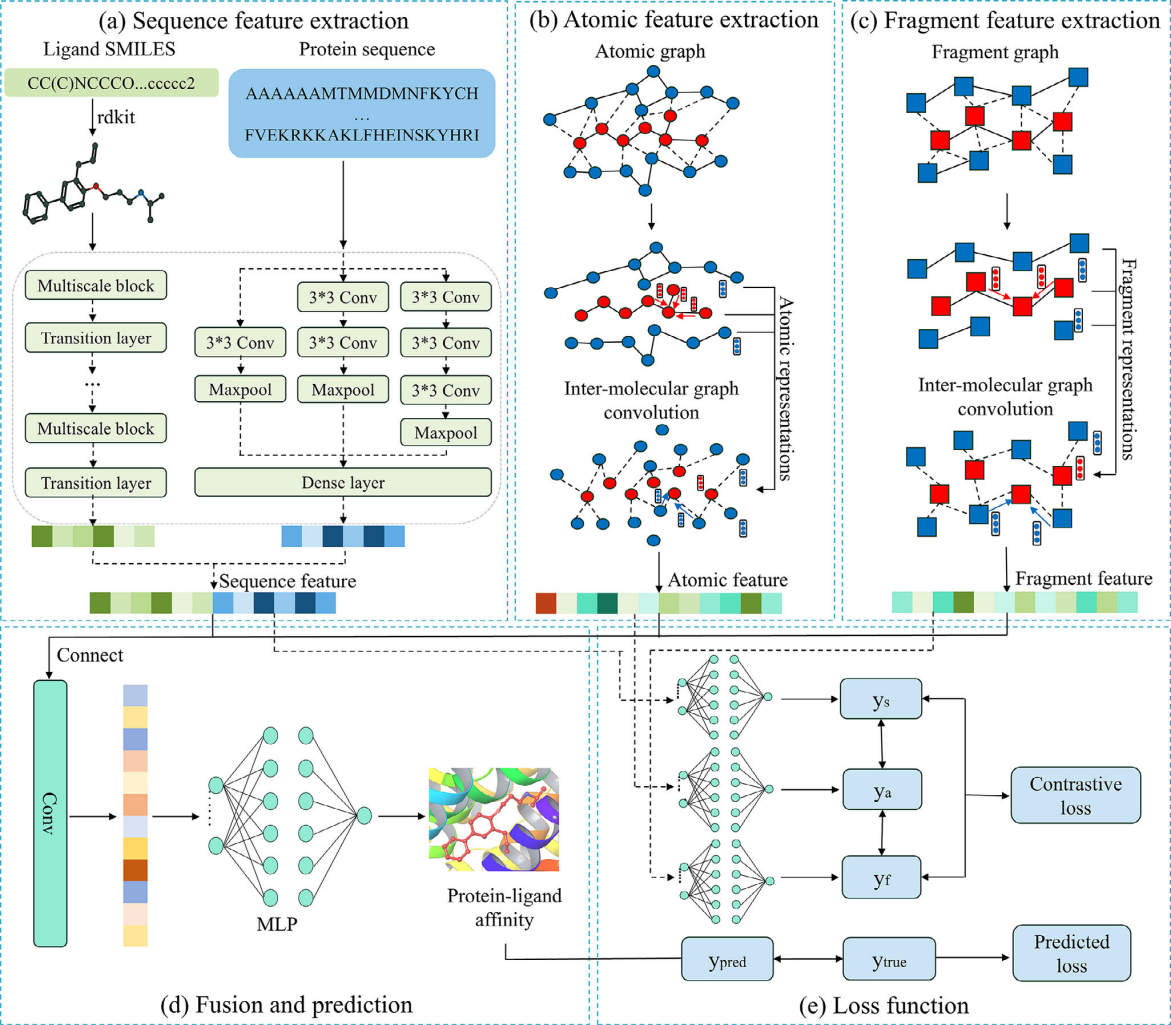
The overall architecture of MF‐Net. The model is composed of five components: (a) sequence feature extraction module, (b) atomic feature extraction module, (c) fragment feature extraction module, (d) fusion and prediction module, and (e) loss function.

### 2.2 Performance Comparison on Binding Affinity Prediction

The performance of MF‐Net on the DTA prediction task was comprehensively evaluated using the PDBBind v2016 dataset, as shown in Table 1. For comparison with previous studies, all models were trained and validated using the same PDBBind benchmark protocol and evaluated on the core set. The performance of the baseline models was taken from their original publications, which followed the same benchmark setting. For MF‐Net, we performed three independent runs with different random seeds to assess the stability of the proposed model, and the results are reported as the mean ± standard deviation. Since repeated‐run statistics were not available for most baseline methods, only the published performance values are reported for comparison.

**TABLE 1:** Comparison results of the proposed MF‐Net and baselines on PDBbind v2016 core set.

Model	RMSE↓	MAE↓	Rp↑	SD↓
DeepDTA [21]	1.443	1.148	0.749	1.445
GAABind [33]	1.298	1.026	0.803	
Pafnucy [28]	1.418	1.129	0.775	1.375
OnionNet [26]	1.278	0.984	0.816	1.257
IGN [27]	1.22	0.94	0.837	
MFE [34]	1.151	0.882	0.851	1.138
EHIGN [25]	1.150(0.022)		0.854(0.004)	
MF‐Net	1.127(0.004)	0.865(0.005)	0.862(0.005)	1.116(0.008)

MF‐Net achieved the best overall performance, with an RMSE of 1.127 ± 0.004, MAE of 0.865 ± 0.005, Rp of 0.862 ± 0.005, and SD of 1.116 ± 0.008. Notably, MFE also exhibits strong performance with an RMSE of 1.151. The superior performance of these multimodal approaches demonstrates that integrating diverse structural and physicochemical features enables the model to more effectively capture complex drug–target interactions. Further comparison reveals that, except for GAABind, which slightly outperforms the interaction‐based Pafnucy in certain cases, most interaction‐based models substantially outperform interaction‐free models. This finding suggests that explicitly modeling structural and physical interactions is essential for accurately predicting binding affinity.

To further evaluate the robustness and generalization capability of MF‐Net under realistic drug discovery scenarios, we constructed three out‐of‐distribution (OOD) evaluation settings while keeping the PDBBind v2016 core set as the test set.

novel ligand split: training ligands share <20% Morgan fingerprint similarity with test ligands.
novel protein split: training proteins share <20% sequence identity with test proteins.
novel complex split: both constraints are applied simultaneously.

The corresponding results are shown in Figure 2. Compared with the standard split, prediction performance gradually decreased as the partitioning setting became more challenging. Under the novel ligand split, RMSE increased to 1.212 ± 0.027. Compared with the other two OOD settings, the performance degradation was relatively limited, suggesting that the hierarchical integration of sequence‐, atomic‐, and fragment‐level representations enables MF‐Net to retain transferable interaction patterns even for ligands with previously unseen structures. A larger performance decline was observed under the novel protein split, where RMSE increased to 1.326 ± 0.029, and Rp decreased to 0.804 ± 0.009. The observed performance decline suggests that sequence divergence alters residue composition and binding‐site characteristics, reducing the transferability of interaction patterns learned from the training data. The novel complex split, which simultaneously excluded both similar proteins and similar ligands, represented the most stringent evaluation scenario. As expected, it yielded the lowest performance, reflecting the cumulative challenge of simultaneously extrapolating across both molecular components. Overall, the model performance gradually decreases as the degree of distribution shift increases. Nevertheless, the performance degradation remained gradual rather than abrupt across the three more difficult OOD settings. This trend suggests that MF‐Net does not rely heavily on memorizing similar training pairs, but instead captures transferable interaction patterns that generalize to unseen protein–ligand complexes.

**FIGURE 2:**
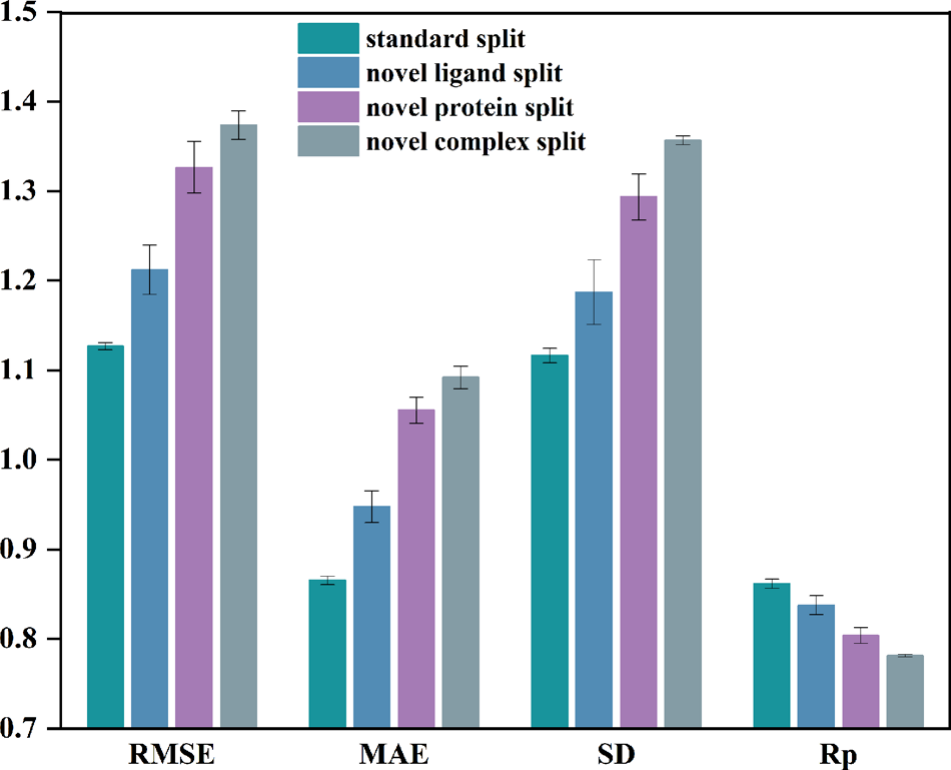
Performance comparison of MF‐Net under the standard split and three out‐of‐distribution evaluation settings on the PDBBind v2016 core set.

### 2.3 Ablation Study

To evaluate the contribution of each component to the overall performance of MF‐Net, we conducted ablation studies under the same experimental settings. Four simplified variants of MF‐Net were implemented:

w/o sequence: removing the sequence feature extraction module.
w/o atomic graph: removing the atomic graph feature extraction module.
w/o fragment graph: removing the fragment graph feature extraction module.
w/o contrastive loss: removing the contrastive loss.

The ablation results on the PDBBind v2016 dataset are shown in Figure 3 and Table 2. MF‐Net consistently achieves the best performance across all evaluation metrics, demonstrating the complementary roles of its individual components in DTA prediction. Specifically, the variant without the sequence feature extraction module exhibited the most significant performance degradation, with RMSE, MAE, and SD increasing by 20.6%, 23.7%, and 19.4%, respectively, and Rp decreasing by 8.5%. This highlights the crucial role of sequence features in providing global biochemical and structural context, which is essential for capturing long‐range dependencies between drug and target. Similarly, removing either the atomic or fragment graph feature extraction module also led to noticeable performance declines, with a greater decline observed when the atomic graph feature extraction module was removed. This result indicates that atomic‐level representations capture fine‐grained physicochemical interactions between the protein pocket and ligand more effectively than fragment‐level features. Finally, the variant without contrastive loss resulted in a 7.3% increase in RMSE and a 7.9% increase in MAE, confirming that the contrastive loss enhances multimodal feature consistency and improves the model's robustness and generalization. Overall, these ablation experiments validate that the synergy among MF‐Net's modules is essential for accurately modeling complex drug–target interactions. The combination of effective multimodal feature fusion and contrastive learning contributes to the superior predictive accuracy and stability of MF‐Net.

**FIGURE 3:**
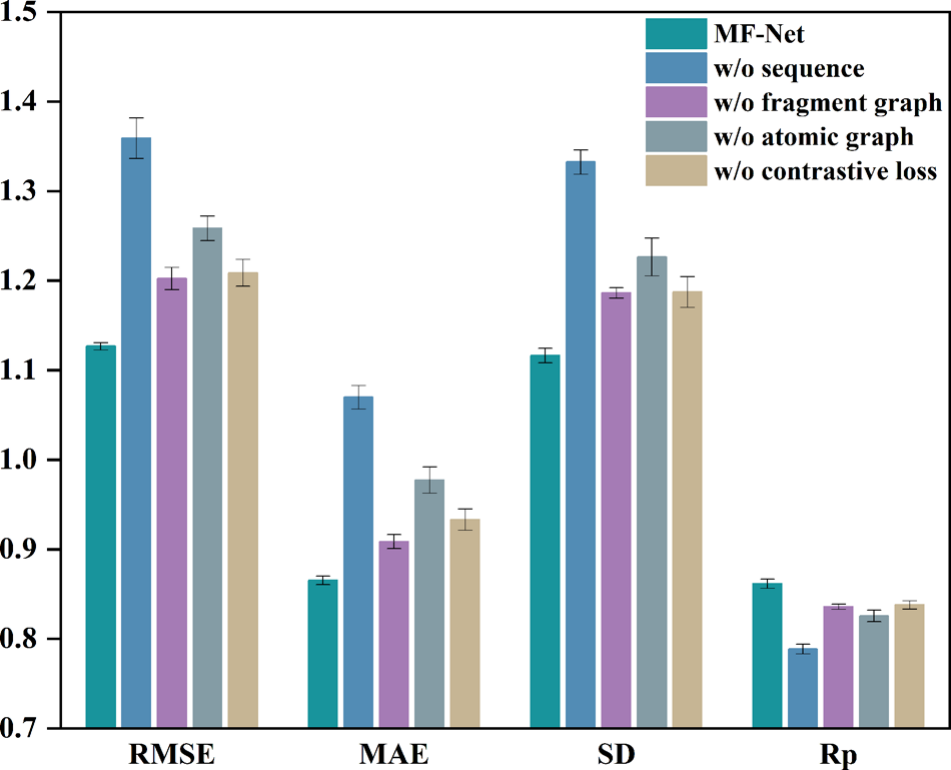
Ablation study results of MF‐Net and the variants on PDBbind v2016 core set.

**TABLE 2:** Ablation study results of MF‐Net and the variants on PDBbind v2016 core set.

Models	RMSE↓	MAE↓	SD↓	Rp↑
w/o sequence	1.359(0.023)	1.070(0.013)	1.332(0.014)	0.789(0.006)
w/o fragment graph	1.202(0.012)	0.909(0.008)	1.186(0.006)	0.836(0.003)
w/o atomic graph	1.258(0.014)	0.977(0.015)	1.226(0.021)	0.826(0.007)
w/o contrastive loss	1.209(0.015)	0.933(0.012)	1.187(0.017)	0.838(0.005)
MF‐Net	1.127(0.004)	0.865(0.005)	1.116(0.008)	0.862(0.005)

To further evaluate the effectiveness of the proposed fusion strategy, we compared MF‐Net with several representative fusion variants, including direct concatenation fusion (w/ concatenation), attention‐based fusion (w/ attention‐based fusion), gated fusion (w/ gated fusion), and transformer‐based fusion (w/ transformer‐based fusion). The ablation results on the PDBBind v2016 dataset are shown in Figure 4 and Table 3.

**FIGURE 4:**
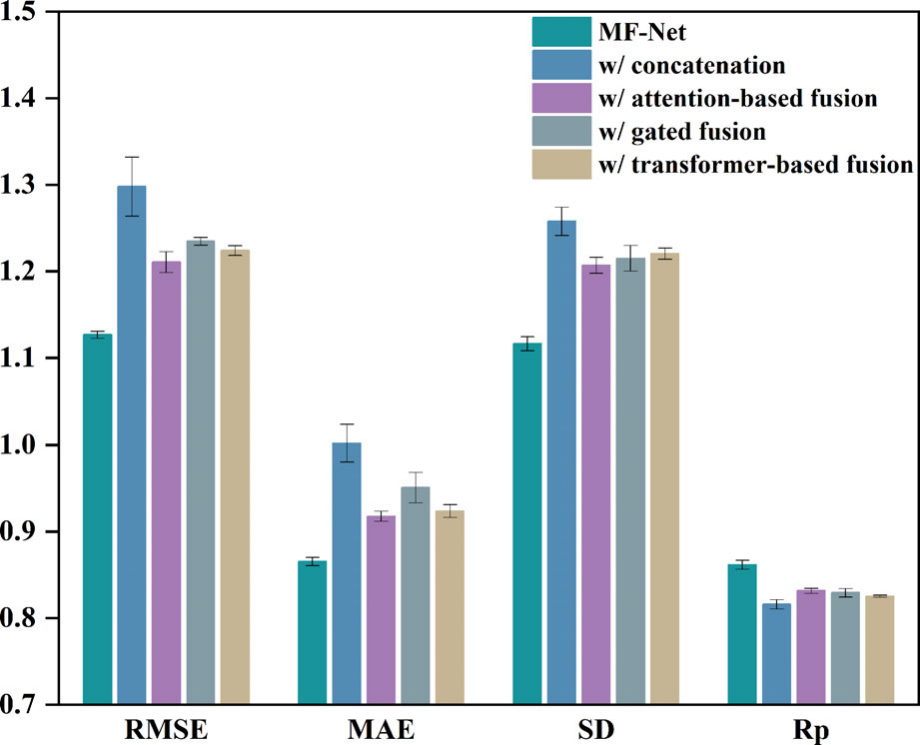
Ablation study results of MF‐Net and the fusion variants on PDBbind v2016 core set.

**TABLE 3:** Ablation study results of MF‐Net and the fusion variants on PDBbind v2016 core set.

Models	RMSE↓	MAE↓	SD↓	Rp↑
w/ concatenation	1.298(0.034)	1.002(0.022)	1.258(0.016)	0.816(0.005)
w/ attention‐based fusion	1.211(0.012)	0.918(0.006)	1.207(0.010)	0.832(0.003)
w/ gated fusion	1.235(0.005)	0.951(0.018)	1.215(0.015)	0.829(0.005)
w/ transformer‐based fusion	1.224(0.006)	0.924(0.007)	1.220(0.006)	0.825(0.001)
MF‐Net	1.127(0.004)	0.865(0.005)	1.116(0.008)	0.862(0.005)

Our experimental results reveal several important insights. Direct concatenation fusion led to substantially degraded prediction performance compared to MF‐Net with the adaptive CNN‐based fusion strategy. Unlike simple concatenation, which directly aggregates heterogeneous features without modeling their relationships, the CNN‐based fusion module captures complementary cross‐modal patterns through shared convolutional kernels, enabling effective integration of sequence‐, atomic‐, and fragment‐level representations. In contrast, attention‐based fusion, gated fusion, and transformer‐based fusion all consistently outperformed direct concatenation fusion, confirming the tangible benefits of adaptive multimodal fusion over simple feature aggregation. Among all evaluated approaches, MF‐Net achieved the best overall performance, yielding the lowest RMSE, MAE, and SD, alongside the highest Rp. Attention‐ and transformer‐based fusion are primarily designed to capture global feature dependencies. Since MF‐Net fuses three modality‐level representations that have already been encoded into compact latent features, the benefits of explicitly modeling long‐range dependencies may be limited in this setting. Gated fusion primarily focuses on adaptive modality weighting and provides less explicit interaction modeling between modalities. These characteristics make the proposed CNN‐based adaptive fusion mechanism suitable for integrating global sequence‐, local atomic‐, and functional fragment‐level information while preserving their complementary contributions. Overall, these ablation results highlight the importance of fusion strategy design in multimodal DTA prediction and suggest that the proposed CNN‐based adaptive fusion module provides an effective approach for multimodal molecular representation learning.

### 2.4 Generic Structure‐Based Virtual Screening

To further evaluate the reliability of MF‐Net, we conducted a generic structure‐based virtual screening task to assess its generalization capability across diverse targets. In this task, models are trained and tested on datasets containing multiple targets to simulate real‐world drug discovery scenarios. For a comprehensive evaluation, MF‐Net was compared with several representative baselines, including two graph neural network‐based methods (IGN and EHIGN), a traditional machine learning approach (RFscore‐VS), and a widely used molecular docking program (Glide‐SP). All machine learning models were trained and validated on the DUD‐E dataset and tested on the DEKOIS2.0 dataset. Notably, four targets (cyp2a6, er‐β, jak3, and pdk1) were excluded from evaluation due to incompatibilities with the feature extraction pipeline of RFscore‐VS.

Figure 5 illustrates the comparative performance of all methods on the generic structure‐based virtual screening task. Overall, the docking tool (Glide‐SP) and the traditional machine learning method (RFscore‐VS) consistently underperformed compared to graph neural network‐based models. This performance gap underscores the superior capacity of deep learning architectures in tasks involving molecular recognition and early enrichment. Notably, MF‐Net outperformed all baselines on early enrichment metrics, attaining state‐of‐the‐art EF0.1% and EF0.5% scores of 29.05 and 23.72, respectively. These results indicate that MF‐Net is particularly effective at prioritizing active compounds when screening a small number of candidates, offering a clear advantage in early‐stage identification of potential lead molecules. However, as the screening threshold increased, IGN and EHIGN achieved better overall performance. This observation suggests that MF‐Net's optimization is particularly biased toward highly active compounds, potentially at the expense of identifying moderately active ones. Such performance behavior can be attributed to MF‐Net's training strategy and contrastive learning design, which emphasize discriminative feature learning for strong binders. The contrastive loss primarily focuses on distinguishing highly active compounds from inactive ones, which inevitably reduces sensitivity toward weakly or moderately active compounds. Although this enhances early enrichment by aligning features across modalities, it also results in a more compact latent space, thereby limiting the model's global search capacity in broader screening scenarios. These findings provide valuable insights for practical virtual screening applications: MF‐Net excels in early enrichment scenarios where identifying top candidates is critical, while models like IGN and EHIGN may be more suitable for large‐scale screening tasks that require broader coverage of active compounds. Therefore, the choice of model should be guided by the specific objectives and practical requirements of the virtual screening scenario to achieve optimal performance.

**FIGURE 5:**
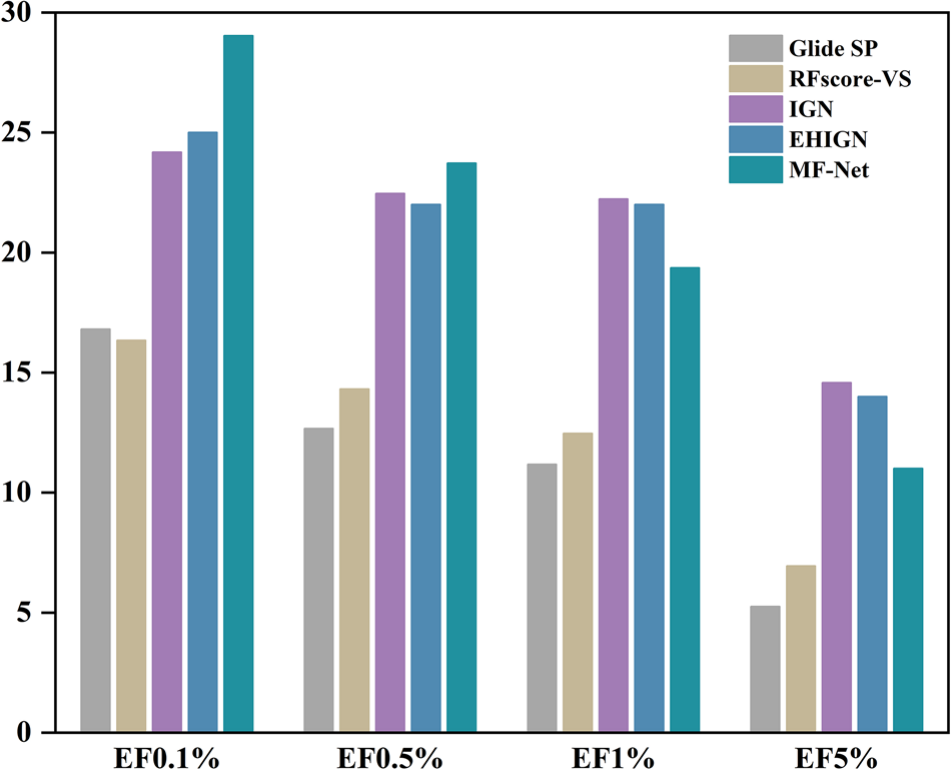
Enrichment factor comparison of the proposed MF‐Net and baselines on DEKOIS2.0 dataset.

### 2.5 Target‐Specific Structure‐Based Virtual Screening

Over the past decade, the DUD‐E dataset has been widely regarded as the gold standard for evaluating the performance of machine learning‐based virtual screening approaches. However, recent studies have revealed that the artificially generated decoys in DUD‐E may introduce hidden biases, potentially leading to an overestimation of predictive accuracy [35, 36]. Therefore, we used the unbiased LIT‐PCBA dataset to evaluate the performance of the MF‐Net model in a target‐specific structure‐based virtual screening task. All the actives and decoys in LIT‐PCBA were experimentally verified and curated under consistent conditions, with high imbalance between actives and decoys to better mimic realistic screening scenarios. MF‐Net was compared with several representative baselines, including traditional docking methods (Surflex and Glide SP), classical machine learning models (RFscore‐VS), and graph neural network‐based methods (IGN and EHIGN). We selected seven representative protein targets (KAT2A, PKM2, FEN1, MAPK1, GBA, ALDH1, and VDR) that each contained more than 100 actives and adopted the original training and test splits defined by the LIT‐PCBA dataset.

Table 4 shows the enrichment factors (EF0.1%, EF0.5%, EF1%, EF5%) of different models on seven representative targets from the LIT‐PCBA dataset. The results demonstrate that traditional docking methods exhibit predictive performance only slightly better than random predictions, especially at early enrichment stages. For instance, Glide SP yields EF0.1% values of 0.00 on KAT2A, PKM2, and FEN1, highlighting the challenges of distinguishing between active and inactive compounds in the LIT‐PCBA dataset. In contrast, AI‐driven methods exhibit markedly superior performance. Notably, the MF‐Net model excels at early enrichment stages, achieving the best performance across all targets at the EF0.1% threshold. For example, on the KAT2A target, its early enrichment ability (EF0.1% = 62.21) far surpasses the second‐best model EHIGN (EF0.1% = 41.47), showing a 50% improvement. Furthermore, MF‐Net attains the best overall performance on five of the seven targets and maintains competitive results even at higher thresholds (EF5%), demonstrating its robustness and generalization across diverse protein targets. The superior performance of MF‐Net can be attributed to its multimodal feature fusion strategy, which integrates sequence, atomic‐graph, and motif‐graph representations to jointly capture both global and local patterns of drug–target interactions. This comprehensive representation enables MF‐Net to generate highly discriminative molecular embeddings, thereby improving screening precision. MF‐Net's remarkable early enrichment capability indicates its strong potential in rapidly identifying high‐affinity compounds at the early stages of virtual screening, which can accelerate the lead identification process.

**TABLE 4:** Enrichment factor comparison of the proposed MF‐Net and baselines on the representative targets from the LIT‐PCBA dataset.

Target	Model	EF0.1%	EF0.5%	EF1%	EF5%
KAT2A	IFP [37]	—	—	3.61	—
	GRIM [37]	—	—	3.61	—
	Surflex [37]	—	—	2.58	—
	Glide SP [27]	0.00	0.00	0.00	0.83
	Rfscore‐VS [27]	0.00	0.00	2.08	1.25
	IGN [27]	0.00	4.16	4.16	1.67
	EHIGN [25]	41.47	16.67	**16.67**	4.58
	MF‐Net	**62.21**	**20.83**	**16.67**	**5.42**
PKM2	IFP [37]	—	—	7.14	—
	GRIM [37]	—	—	5.86	—
	Surflex [37]	—	—	0.18	—
	Glide SP [27]	0.00	0.00	0.00	0.97
	Rfscore‐VS [27]	8.30	1.66	1.66	1.83
	IGN [27]	0.00	3.32	2.50	3.50
	EHIGN [25]	**21.78**	7.33	8.08	4.56
	MF‐Net	**21.78**	**14.66**	**9.55**	**4.85**
FEN1	IFP [37]	—	—	6.78	—
	GRIM [37]	—	—	7.32	—
	Surflex [37]	—	—	4.34	—
	Glide SP [27]	0.00	2.17	2.17	4.13
	Rfscore‐VS [27]	65.05	26.08	14.13	5.22
	IGN [27]	119.26	41.29	30.42	10.65
	EHIGN [25]	154.57	**75.41**	**52.18**	**14.22**
	MF‐Net	**209.77**	66.54	42.19	13.33
MAPK1	IFP [37]	—	—	1.62	—
	GRIM [37]	—	—	3.90	—
	Surflex [37]	—	—	1.62	—
	EHIGN [25]	37.96	12.98	9.08	3.37
	MF‐Net	**50.61**	**18.17**	**11.68**	**4.41**
GBA	IFP [37]	—	—	9.64	—
	GRIM [37]	—	—	10.84	—
	Surflex [37]	—	—	8.43	—
	EHIGN [25]	96.87	38.96	26.82	7.80
	MF‐Net	**121.05**	**48.69**	**29.25**	**9.27**
ALDH1	IFP [37]	—	—	15.35	—
	GRIM [37]	—	—	**15.76**	—
	Surflex [37]	—	—	0.96	—
	EHIGN [25]	14.99	12.14	9.90	5.49
	MF‐Net	**20.24**	**13.64**	11.84	**6.13**
VDR	IFP [37]	—	—	1.24	—
	GRIM [37]	—	—	1.24	—
	Surflex [37]	—	—	0.00	—
	EHIGN [25]	**38.70**	20.77	**18.82**	**7.01**
	MF‐Net	**38.70**	**22.07**	16.88	6.88

### 2.6 Visualization Results

To better reveal the contribution of each modality to the final prediction, we visualized the importance of ligand components for the drug–target complex with PDB ID 2V3E. As shown in Figure 6, the color intensity reflects the relative importance of each molecular component under different modalities, with darker colors indicating higher importance. The drug–target interactions were identified using Schrödinger software (version 2019) and illustrated in a two‐dimensional interaction map (Figure 6e). The results show that four hydroxyl groups on the ligand form hydrogen bonds with residues TRP179, TRP381, ASN234, ASP127, GLU340, and ASN396. These hydrogen bonds not only provide binding energy for the drug–target interaction but also precisely anchor the ligand within the binding pocket. In addition, the amino group in the ligand forms salt bridges with GLU235 and GLU340, and engages in cation‐π interactions with TYR313. The cooperative effects of hydrogen bonds, salt bridges, and cation‐π interactions jointly contribute to the stability of ligand binding. Collectively, these noncovalent interactions play a critical role in determining the overall binding affinity.

**FIGURE 6:**
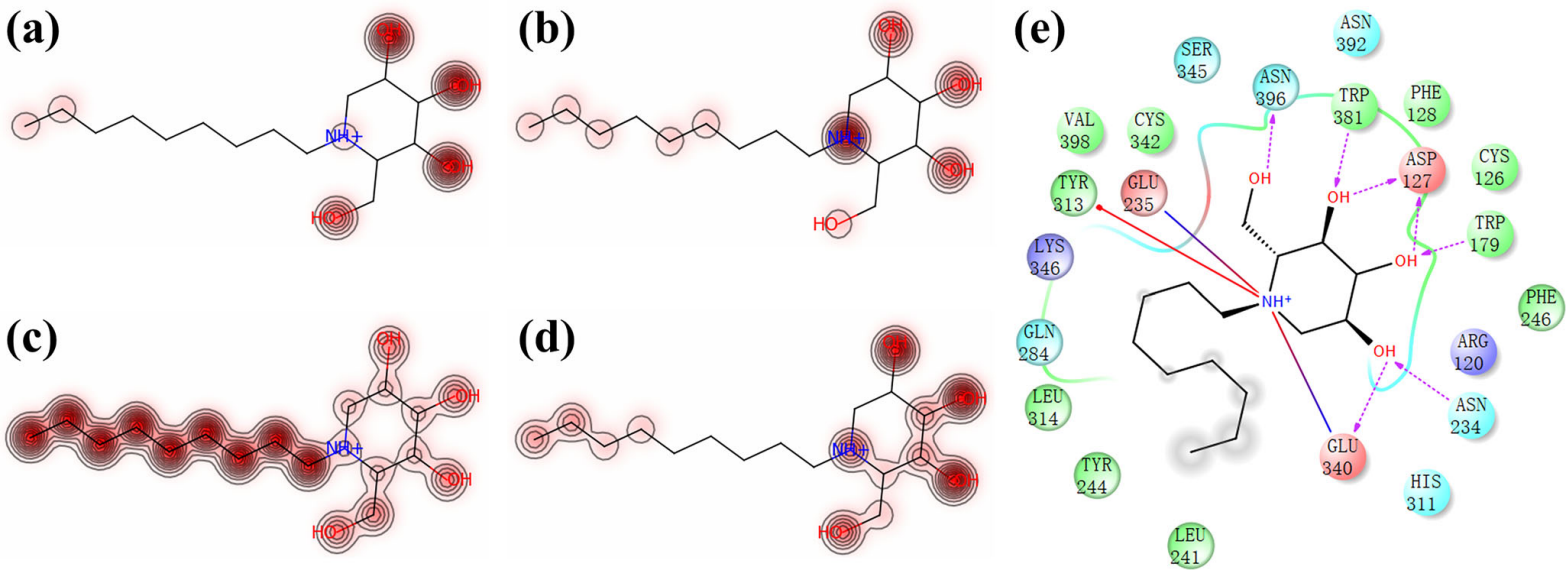
Visualization of key ligand features in crystal structure 2V3E from the Protein Data Bank (PDB). (a) Visualization of atom‐level importance based on sequence features. (b) Visualization of atom‐level importance based on atomic graph features. (c) Visualization of atom‐level importance based on fragment graph features. (d) Visualization of atom‐level importance based on fusion features. (e) The 2D binding mode of HPK1‐ligand (2V3E).

The visualization results further show that the three modalities of sequence, atomic graph, and fragment graph are capable of capturing key drug–target interactions such as hydrogen bonds, salt bridges, hydrophobic interactions, and cation‐π interactions. However, their attention to different molecular components varies, suggesting that each modality captures distinct interaction patterns. Specifically, the regions assigned high importance by the sequence modality frequently overlapped with those ligand atoms that are known to form hydrogen bonds in the corresponding experimentally determined complex structures. This observation suggests that the sequential patterns and evolutionary conservation information captured by the sequence representation may statistically correlate with binding‐relevant positions that tend to participate in key noncovalent interactions upon complex formation. This observation is also consistent with the ablation results, where removing the sequence modality leads to the largest performance degradation. The atomic graph modality effectively captures atomic‐level local chemical environments, assigning high importance to atoms involved in key noncovalent interactions, including hydrogen bonds, salt bridges, and cation–π interactions. In contrast, the fragment graph modality effectively captures functional domains but shows less clarity in assigning interaction priorities. By integrating sequence, atomic graph, and fragment graph modalities, the model not only simultaneously captures diverse interaction types but also emphasizes the most critical ones, such as hydrogen bonding. Overall, the three modalities characterize drug–target interactions at complementary levels, and their fusion enables a more comprehensive understanding of the complex interactions between drugs and targets.

### 2.7 Case Study

To further evaluate the practical effectiveness of MF‐Net in real‐world settings, we conducted a structure‐based virtual screening to identify potential inhibitors targeting HPK1. HPK1 is predominantly expressed in hematopoietic cells and functions as a negative regulator of T‐cell and B‐cell signaling. Inhibition of HPK1 activity has been shown to enhance the cytotoxic activity of immune cells against tumor cells, making it a promising target in tumor immunotherapy. To avoid potential data leakage and ensure a fair evaluation of generalization capability, MF‐Net was trained on the 3DKKIBA dataset without incorporating any HPK1‐related data. 3DKKIBA is constructed by extracting high‐confidence kinase domains and key binding sites from 3D structures predicted by AlphaFold2 [38]. The trained model was then applied to screen approximately 200,000 compounds from the ChemDiv library against HPK1. In parallel, molecular docking was performed using Schrödinger software, where all compounds were docked into the ATP‐binding pocket of HPK1 (PDB ID: 7R9N) and ranked according to docking scores. The top 2000 compounds based on docking scores and the top 2000 compounds predicted by MF‐Net were intersected, resulting in 286 candidate molecules. To maximize structural diversity, the selected compounds were further grouped using k‐means clustering. Twenty representative compounds were then chosen based on both predicted binding affinities and careful visual inspection of binding modes, with particular emphasis on key interactions involving residues Glu92 and Cys94 in the hinge region [32], which are critical for kinase‐ligand binding. Subsequently, the selected compounds were experimentally evaluated using the ADP‐Glo kinase activity assay at a concentration of 50 µM, with Sunitinib included as a positive control. Among the 20 tested compounds, seven candidates (designated HQ2026‐01 to HQ2026‐07) exhibited inhibition rates exceeding 80%. Their IC_50_ values were further determined using a ten‐point, four‐fold serial dilution assay.

As shown in Figure 7a, the identified compounds exhibit substantial structural diversity, encompassing distinct core scaffolds and substitution patterns. Notably, HQ2026‐01 achieved near‐complete inhibition at 50 µM, comparable to the reference inhibitor Sunitinib (Figure 7b), and displayed similar potency with an IC_50_ value of 5.63 nM (5.44 nM for Sunitinib) (Figure 7c). Several compounds, including HQ2026‐03, HQ2026‐04, HQ2026‐05, and HQ2026‐06, exhibited inhibition rates exceeding 90% at 50 µM, indicating strong activity across multiple chemotypes. Among them, HQ2026‐03 demonstrated the highest potency, with an IC_50_ value of 0.41 nM, surpassing that of the reference inhibitor. Interestingly, several of the identified hits correspond to known kinase inhibitors, including HQ2026‐02 (a PDGFR inhibitor), HQ2026‐04 (Torkinib, an mTOR inhibitor), and HQ2026‐06 (Entrectinib, an approved therapeutic for nonsmall cell lung cancer). This observation is consistent with the well‐established polypharmacology of kinase‐targeting compounds, where cross‐reactivity across kinase families is frequently observed. Rather than diminishing the significance of these findings, the rediscovery of such biologically active molecules highlights the ability of MF‐Net to effectively prioritize functionally relevant chemotypes and suggests its potential utility in drug repurposing. Furthermore, although HQ2026‐07 showed relatively weaker activity (IC_50_ = 5697 nM), its distinct chemical scaffold may provide a promising starting point for subsequent structure optimization.

**FIGURE 7:**
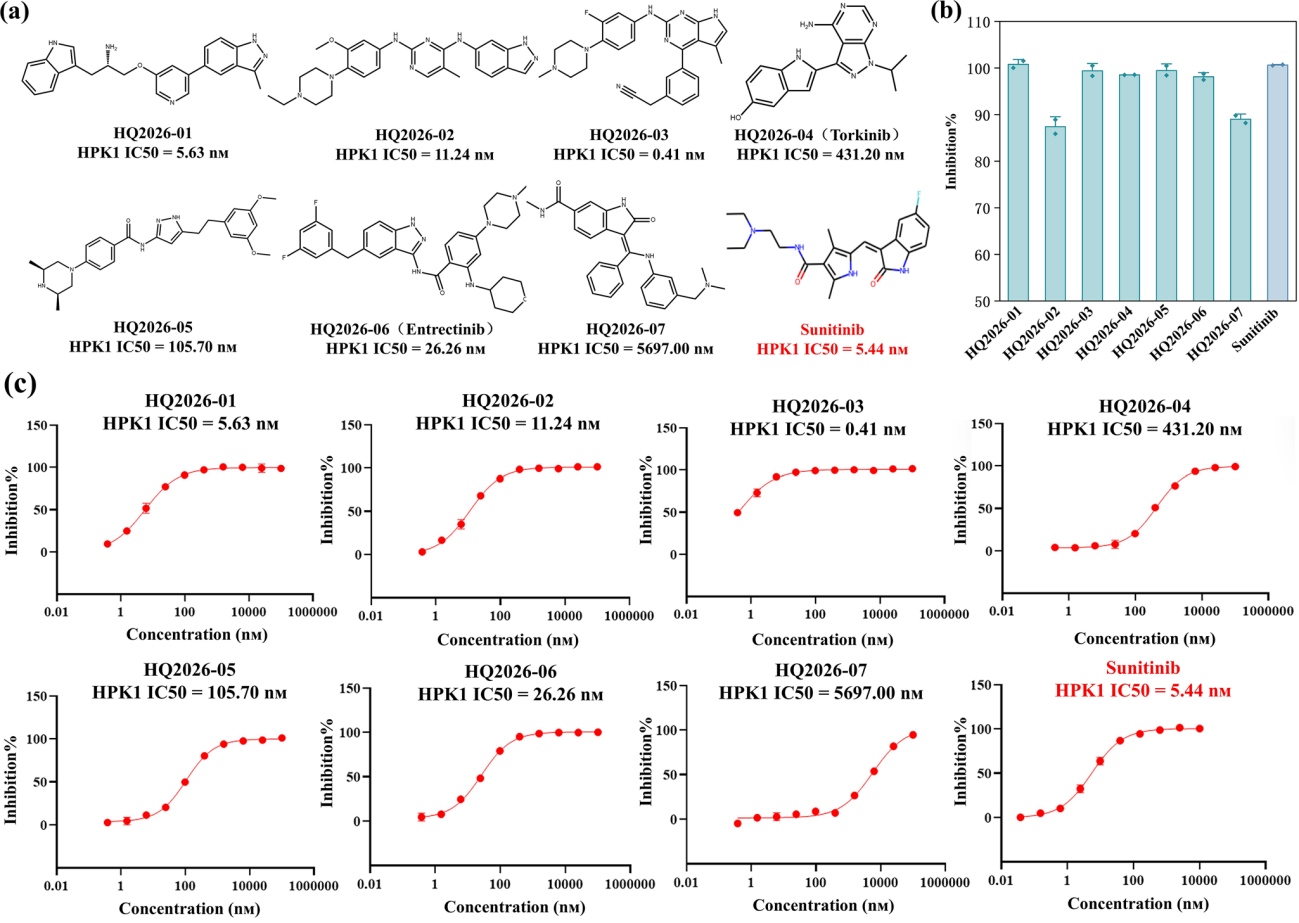
The chemical structures of seven MF‐Net‐identified compounds and the positive control, along with their inhibition rates at a concentration of 50 µM and their IC_50_ values. (a) Chemical structures of seven candidate compounds exhibiting greater than 80% inhibition at 50 µM and the positive control. (b) Inhibitory ratio of seven candidate compounds and the positive control at 50 µM concentration. (c) IC_50_ values of seven candidate compounds and the positive control against HPK1.

## 3 Conclusions

In this study, we propose MF‐Net, a novel multimodal fusion framework for DTA prediction that unifies both interaction‐free and interaction‐based representations to achieve a comprehensive understanding of drug–target interactions. By integrating sequence, atomic graph, and fragment graph features, MF‐Net effectively captures global contextual information, local atomic interactions, and high‐level functional relationships across multiple molecular scales. The model introduces several key innovations, including fingerprint‐guided fragment initialization, which combines prior chemical knowledge with learnable representations, an adaptive feature fusion mechanism that dynamically balances modality contributions, and a contrastive alignment strategy that enhances cross‐modal consistency and robustness to heterogeneous data.

Extensive experiments on multiple benchmarks demonstrate the superiority and generalizability of MF‐Net. On the PDBBind v2016 dataset, MF‐Net achieves state‐of‐the‐art performance in DTA prediction. Across virtual screening evaluations on the DUD‐E, DEKOIS2.0, and unbiased LIT‐PCBA datasets, MF‐Net consistently demonstrates strong early enrichment capability and robust generalization performance. Furthermore, the ablation studies confirm that each modality plays a vital role in improving predictive accuracy, and the visualization analyses reveal MF‐Net's strong ability to capture biologically meaningful interaction patterns. To further validate its real‐world applicability, MF‐Net was employed in the virtual screening of potential HPK1 inhibitors. The model successfully identified seven highly active candidates, demonstrating its practical utility in structure‐based drug discovery. Overall, MF‐Net not only advances the accuracy and interpretability of DTA prediction but also provides a robust, multiscale, and biologically grounded representation framework for intelligent drug discovery and virtual screening applications.

While MF‐Net demonstrates strong performance across multiple benchmarks, several limitations remain that highlight opportunities for future research. First, the training data are limited by the coverage and quality of experimentally determined complexes, which may introduce dataset bias. In addition, although MF‐Net successfully identified several potent HPK1 inhibitors with nanomolar activity, the current study does not establish target selectivity or HPK1‐specific molecular recognition. Future studies will extend the experimental validation of the identified HPK1 inhibitors through kinase selectivity profiling, orthogonal binding assays, and independent reproducibility assessment. Given the conserved nature of kinase ATP‐binding pockets and the polypharmacology commonly observed among kinase inhibitors, these studies will help characterize target selectivity, clarify HPK1‐specific recognition mechanisms, and support the further development of the identified compounds.

## 4 Methods

### 4.1 Datasets

In this study, we conducted two types of experiments: DTA prediction (regression task) and large‐scale structure‐based virtual screening (classification task). For the DTA prediction, we used the PDBBind v2016 dataset [39, 40] as the benchmark. The PDBBind dataset contains experimentally determined DTA data for biomolecular complexes derived from the Protein Data Bank [41], with affinity values reported as K_i_, K_d_, and IC_50_. Following the standard data splitting protocol in previous studies [34, 42], 1000 drug–target complexes were randomly selected from the refined set to construct the validation set, while the remaining complexes from the refined and general sets (10,451 samples in total) were used for training. The core set (250 samples) was used as the test set, and all overlapping entries were removed from the training and validation sets to avoid data leakage.

For the structure‐based virtual screening tasks, we employed three widely used benchmark datasets: DUD‐E [43], DEKOIS2.0 [44], and LIT‐PCBA [45]. The DUD‐E dataset includes 102 targets from diverse protein families and 22,886 active compounds. Each active compound is paired with approximately 50 decoys that share similar physicochemical properties but differ in 2D topological structures. The DEKOIS2.0 dataset includes 81 structurally diverse targets, each containing 40 active compounds and 1,200 decoys. To ensure objective evaluation and mitigate dataset‐specific overfitting, MF‐Net was trained and validated on the DUD‐E dataset (92883 samples) and tested on the DEKOIS2.0 dataset (28,334 samples). This cross‐dataset validation strategy provides a more reliable measure of generalization across structurally distinct datasets. The distributions of protein and ligand sequence lengths across the PDBBind v2016, DUD‐E, and DEKOIS2.0 datasets are shown in Figure S1. The LIT‐PCBA dataset is an unbiased dataset constructed using the Asymmetric Validation Embedding strategy [46] to eliminate potential biases between datasets. The 3D binding structures of the LIT‐PCBA dataset were derived from Shen et al. [47], whose consider 7 targets with more than 100 active compounds.

### 4.2 Baselines

We compared MF‐Net with a set of representative baseline models. For the DTA prediction task, the baseline models can be broadly classified into two categories: interaction‐free models and interaction‐based models. Interaction‐free models process drug and protein features independently without explicit interaction modeling. For example, DeepDTA [21] employs two CNNs to extract features separately from protein amino acid sequences and ligand SMILES strings, then predicts binding affinity by concatenating these features. Meanwhile, GAABind [33] incorporates a geometry‐aware attention mechanism within a multitask learning framework to enhance prediction accuracy by modeling spatial geometric relationships. On the other hand, interaction‐based models explicitly capture binding dynamics by modeling structural and physicochemical interactions within drug–target complexes. For example, Pafnucy [28] represents drug–target complexes as 3D voxel grids and employs CNNs to extract spatial features for binding affinity prediction. OnionNet [26] quantifies interatomic contacts at multiple distance thresholds, improving the interpretability of binding mechanisms through physically meaningful interaction patterns. IGN [27] decomposes the drug–target complex into ligand, protein, and interaction graphs, leveraging two GCNs to model intramolecular and intermolecular interactions separately. MFE [34] integrates protein surface, 3D structural, and sequential information via a cross‐attention mechanism, ensuring feature alignment across modalities. Its representations are extracted from parallel branches and fused at the feature level, without explicit hierarchical modeling across different molecular scales. EHIGN [25] employs a heterogeneous GNN to distinguish covalent and noncovalent interactions, followed by an interpretable atom‐pair affinity accumulation mechanism for binding strength prediction.

To further validate MF‐Net's performance in the virtual screening task, we conducted comparative analyses against both traditional machine learning approaches and classical molecular docking tools. RFscore‐VS is a random forest‐based approach that employs engineered features (e.g., atomic pair distances and hydrogen bonding patterns) for efficient large‐scale virtual screening. Surflex utilizes molecular surface shape complementarity and empirical force field scoring functions to support flexible docking and efficient virtual screening. Glide‐SP is a standard‐precision mode of the Glide molecular docking software developed by Schrödinger. It first generates candidate conformations rapidly and then refines them using force‐field optimization and scoring, achieving an effective balance between computational efficiency and prediction accuracy.

### 4.3 Multimodal Input

To comprehensively characterize drug–target complexes, MF‐Net employs a unified multimodal input strategy that integrates interaction‐free and interaction‐based representations, as illustrated in Figure 8. The strategy represents the complex from three hierarchical levels (sequence, atomic graph, and fragment graph) to capture global structural context, local chemical environments, and high‐level functional interactions.

**FIGURE 8:**
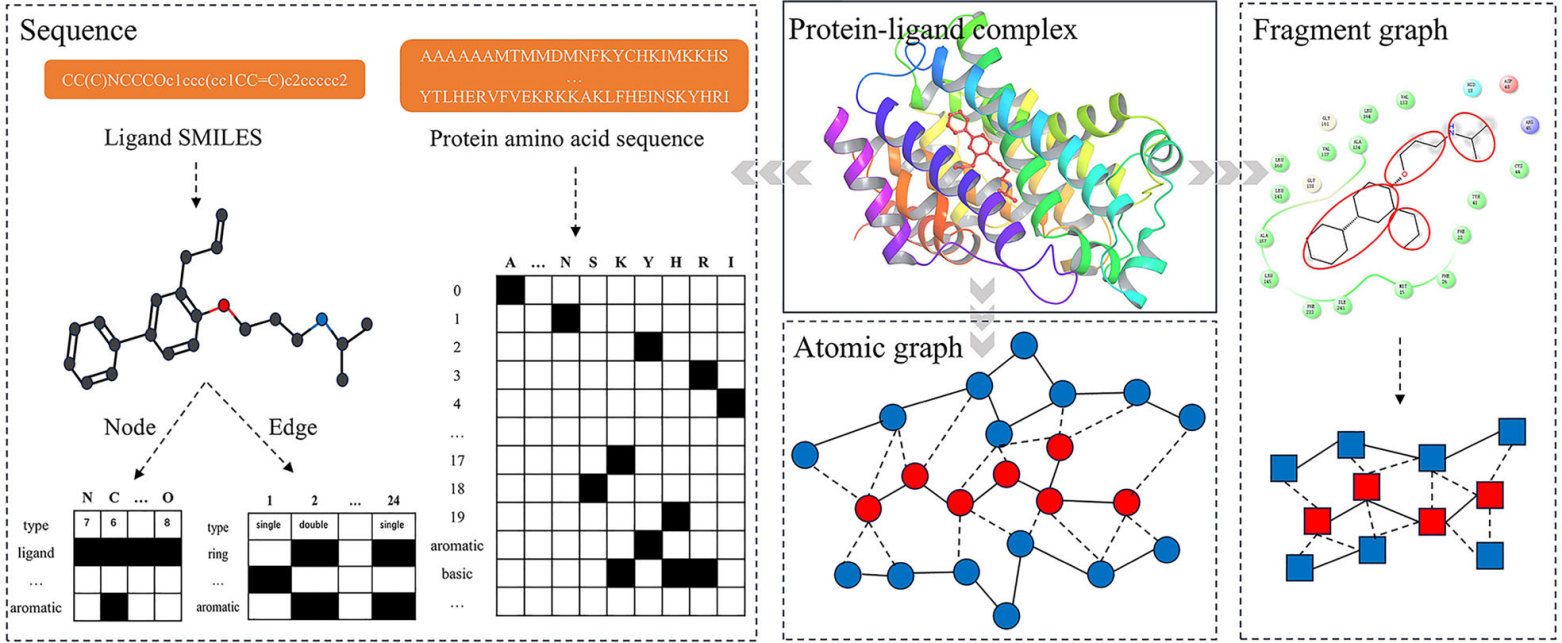
Multimodal input representation of drug–target complexes. Drug–target complexes are encoded using three complementary modalities: sequence, atomic graph, and fragment graph.

The sequence representation takes the amino acid sequence of the protein and the SMILES string of the ligand as input. Each amino acid is encoded as a one‐hot vector based on residue type, augmented with physicochemical descriptors derived from the R‐group (e.g., polarity, molecular weight, acidity/basicity, and aromaticity), resulting in a 33‐dimensional residue feature vector. The ligand SMILES string is converted into a molecular graph using RDKit, where nodes represent atoms and edges represent chemical bonds. As shown in Table 5, each atom is represented by a 36‐dimensional feature vector, including atom type, formal charge, hybridization, electronic, aromaticity, etc. By integrating protein sequence and ligand graph features, the model learns a global representation that captures the overall structural context of the drug–target pair and provides global information for subsequent local and functional‐level modeling.

**TABLE 5:** Atom features are used in the atomic graph and the ligand graph.

Feature	Description	Values	Length
Node features			
Atom type	Type of atom	C,N,O,F,P,S,Cl,Br,I	9
Degree	Number of heavy atom neighbors	0,1,2,3,4,5	6
Formal charge	The electronic charge assigned to the atom	−1, ‐2, 1, 2, 0	5
Chirality	The chirality type of the atom	0, 1, 2, 3	4
Number Hs	Number of bonded hydrogen atom	0, 1, 2, 3, 4	5
Hybridization	The hybridization form of the atom	SP, SP2, SP3, SP3D, SP3D2	5
Aromaticity	The atom is or is not part of an aromatic system	0,1	1
Protein	Whether the atom belongs to a protein	0,1	1
Edge features			
Bond type	Type of bond	Single, double, triple, aromatic, others	5
Aromaticity	The atom is or is not part of an aromatic system	0,1	1
Ring	The bond is or is not part of a ring	0,1	1
Protein	Whether the atoms at both ends of the bond belong to the protein	0,1	1
Ligand	Whether the atoms at both ends of the bond belong to the ligand	0,1	1
Distance	Bond distance	0.348, 0.253, …	1

To capture fine‐grained local interactions, the atomic‐level representation focuses on the binding pocket region, where both protein and ligand are modeled as atomic graphs. Atoms are defined as nodes, while chemical bonds and spatial proximity relationships are defined as edges. Similar to the ligand graph of sequence representation, the node features are represented as 36‐dimensional vectors and edge features are represented as 10‐dimensional vectors, including bond type and interatomic distance, as shown in Table 5. Cross‐molecular edges are constructed if the distance between a protein atom and a ligand atom is less than 5.0 Å, capturing essential noncovalent interactions such as hydrogen bonds and van der Waals forces. This atomic graph provides detailed information about the local chemical environment and spatial arrangement of atoms within the binding site, laying a solid foundation for subsequent interaction modeling.

To capture higher‐level structural information, we further constructed a fragment graph that focuses on the interactions between amino acid residues in the protein binding pocket and functional motifs in the ligand. Amino acid residues are defined as nodes in the protein pocket fragment graph, and their features are consistent with those used in the sequence representation. Ligand molecules are decomposed into chemically meaningful motifs using the BRICS algorithm, with additional cleavage of nonring bonds connected to ring structures to obtain finer substructures. Each node in the ligand motif graph is encoded as a 194‐dimensional feature vector combining MACCS keys and pharmacophore fingerprints, which describe substructure patterns and functional group distributions. Edges between protein residues and ligand motifs are constructed when their spatial distance is less than 8.0 Å. This fragment graph emphasizes functional‐domain interactions and provides higher‐level structural information that complements the atomic graph. The distance thresholds of 5 and 8 Å were adopted following previous studies [27] and provide a reasonable trade‐off between graph sparsity and interaction coverage.

### 4.4 Sequence Feature Extraction Module

In the sequence feature extraction module, the MF‐Net framework integrates hierarchical information from both the protein sequence and the ligand graph to generate multilevel representations. It comprises two components: a multiscale convolutional neural network (MCNN) for protein sequences and a multiscale graph neural network (MGNN) for ligand graphs. All protein sequences were padded or truncated to a fixed length before feature extraction. The MCNN architecture integrates three parallel convolutional branches with different kernel sizes to capture residue interactions at various scales, reflecting both short‐range (e.g., hydrogen bonding) and long‐range (e.g., hydrophobic) dependencies. After the convolutional operation, a pooling layer is applied to generate fixed‐length feature vectors. These multiscale features are concatenated and fed into a fully connected layer to obtain the protein sequence representation *R_p_*.

(1) $$\;R_{P}=W\;cat\left(M\left(C_{1}\left(f_{p}\right)\right),\;M\left(C_{2}\left(f_{p}\right)\right),\;M\left(C_{3}\left(f_{p}\right)\right)\right)$$

where *f_p_* denotes the initial feature of the protein sequence, and *C_i_* denotes a convolutional branch with *i* convolution layers that map *f_p_* into a matrix. *M* denotes the max‐pooling operation, *cat*( ) denotes simple concatenation, and *W* is a learnable matrix.

The MGNN extracts atomic interactions within ligand graphs through three densely connected multiscale blocks, each followed by a transition layer. Each block contains *n* graph convolutional layers, where outputs from all preceding layers are directly connected to subsequent layers to mitigate gradient vanishing. Through this dense connectivity, the node representation at each layer implicitly encodes information from progressively larger subgraph neighborhoods—ranging from 1‐hop local chemical environments (e.g., immediate bond partners and functional groups) to 2‐hop and higher‐order topological contexts (e.g., ring systems, conjugated pathways, or distant substituent effects). By concatenating the outputs from all intermediate layers, the final node representation preserves multiscale structural patterns, enabling the model to simultaneously capture fine‐grained local chemistry and broader molecular topology. Next, the hidden state of each node is iteratively updated based on the features of its neighboring nodes, and node‐level batch normalization (BN) is applied to stabilize training.

(2) $$\begin{array}{ccc} \;f_{l} & = & BN\left(W_{1\;}cat\left(f_{l}^{0},\;f_{l}^{1},\text{…},\;f_{l}^{n}\;\right)\right.\\ & & +\left.W_{2\;}\sum_{j\in N\left(l\right)}cat\left(f_{j}^{0},\;f_{j}^{1},\text{…},\;f_{j}^{n}\right)\right) \end{array}$$

where *W*_1_ and *W*_2 _ are learnable matrices, and *N*(*l*) denotes the neighbors of node *l*.

A global aggregation operation is then applied to generate a comprehensive feature vector for the ligand graph *R_L_*.

(3) $$\;R_{L}=\frac{1}{\left|L\right|}\sum_{l\in L}f_{l}$$

where |*L*|is the number of nodes in the ligand graph.

Finally, the sequence features were obtained by the concatenation of the protein sequence features and the ligand graph features.

(4) $$\;R_{s}=cat\left(R_{P},R_{L}\right)$$

### 4.5 Atomic Feature Extraction Module

In the atomic graph feature extraction module, we integrate SchNet with an intermolecular graph convolution module to characterize atomic‐level interactions between drugs and targets. SchNet effectively captures interatomic relationships through distance encoding, continuous‐filter convolutions, and message passing. For each edge in the atomic graph, the spatial distance between atoms is computed and encoded using a Gaussian radial basis function, which maps continuous distance values into a discrete feature space.

(5) $$\;e_{ij}=G\left(\left|\left|p_{i}-p_{j}\right|\right|\right)$$

where *p_i_* and *p_j_* denote the spatial coordinates of node *i* and node *j* respectively, and *G* denotes the Gaussian radial basis function.

Next, rotationally invariant continuous‐filter convolution (cfconv) is employed to learn atomic interactions by applying distance‐dependent filters, ensuring invariance to the rotation of the atomic graph. Specifically, the feature of each neighboring atom *x_j_* is multiplied by the distance‐dependent convolution kernel *e_ij_* and propagated to atom *i*. The aggregated messages are combined with the original node features through residual connections to stabilize gradients and enhance feature refinement.

(6) $$\;x_{i}=x_{0}+\sum_{j\in N\left(i\right)}e_{ij}\cdot x_{j}$$

where *x*_0_ and *x_i_* denote the original and updated features of atom *i*, and *N*(*i*) denotes the neighbors of atom *i*.

The updated node features are then fed into the intermolecular graph convolution module, which captures pairwise atomic interactions between protein and ligand atoms. The updated node features are concatenated with the initial edge features $e_{pl}^{0}$ of drug–target pairs. These combined features are then input into an MLP, followed by edge‐level batch normalization to facilitate stable and efficient training of the deep network.

(7) $$\;e_{pl}=BN\left(MLP\left(cat\left(e_{pl}^{0},\;x_{i}\right)\right)\right)$$

where, $e_{pl}^{0}$ denotes the initial edge feature of the protein atom and the ligand atom.

Finally, the global atomic graph representation is obtained by combining weighted sum pooling and max‐pooling operations. The weighted sum pooling captures overall structural information, while max pooling highlights the most informative features.

(8) $$\;R_{A}=cat\left(\sigma \left(W_{e}\cdot e_{pl}\right)\cdot e_{pl},\;M\left(e_{pl}\right)\right)$$

where σ denotes the tanh activation function, *W_e_* is the learnable matrix, and *M* denotes the max‐pooling operation.

### 4.6 Fragment Feature Extraction Module

In the fragment graph feature extraction module, a hybrid strategy combining a context‐aware CFTransformer and an intermolecular graph convolution network is employed to capture high‐level interactions between protein residues and ligand motifs. The atomic graph feature extraction module focuses on atom‐level spatial dependencies, while this module emphasizes functional and structural interactions at the fragment level. The spatial relationships between fragment nodes are first encoded using a Gaussian radial basis function to generate edge features *e_ij_*, as defined in Equation 5. The CFTransformer then iteratively updates fragment node representations through multihead attention blocks. In each block, normalized node features are projected into query, key, and value spaces through independent convolutional operations.

(9) $$Q=Conv\left(LN\left(x\right)\right)$$

(10) $$K=Conv\left(LN\left(x\right)\right)$$

(11) $$V=Conv\left(LN\left(x\right)\right)$$

Attention weights are then computed based on the similarity between query and key vectors, modulated by the corresponding edge features. Attention calculations are performed in parallel across multiple heads, enhancing the model's ability to learn complex patterns.

(12) $$\;\alpha_{ij}=\frac{Q_{i}\cdot K_{j}}{\sqrt{e_{ij}}}$$

where *Q_i_* denotes the query vector of node *i*, *K_j_* denotes the key vector of node *j*, and *e_ij_* denotes the edge feature between node *i* and node *j*.

Each node aggregates messages from its neighboring nodes according to the learned attention weights. Specifically, for each node, the learned attention weight α_*ij*_ is used to weight the value vector *V_j_* of each neighboring node *j*. The weighted features from all neighboring nodes are then aggregated to form the message representing the local interaction context of node *i*. The aggregated message is combined with the original node feature *x*_0_ through a residual connection and refined using an MLP.

(13) $$\;x_{i}=MLP\left(x_{0}+\sum_{j\in N\left(i\right)}\alpha_{ij}\cdot V_{j}\right)$$

Subsequent operations follow the same procedure as in the atomic feature extraction module. The refined fragment features are fed into the intermolecular graph convolution network, and the final fragment‐level representation *R_F_* is obtained through a combined weighted sum and max pooling, as defined in Equations 7 and 8.

### 4.7 Fusion and Prediction Module

The core challenge of multimodal models lies not only in constructing diverse modalities but also in effectively integrating them. Multimodal fusion aims to select, extract, and combine complementary information from heterogeneous data sources to obtain a more comprehensive feature representation. For each drug–target pair, we extracted sequence features, atomic interaction graph features, and fragment interaction graph features as described in previous sections. In the fusion and prediction module, these three features are concatenated and fed into a CNN for further processing. The CNN automatically extracts features through its convolutional layers and captures local relationships between different modalities. The lower convolutional layers focus on learning key features of each modality, while the higher layers integrate them into a unified global representation. The fused multimodal features are subsequently fed into an MLP with ReLU activation functions to generate the final prediction results.

### 4.8 Loss Function

Traditional methods typically rely on supervised losses from downstream tasks to guide model training. However, the representations learned from different modalities may exhibit substantial discrepancies in distribution and scale due to the inherent heterogeneity of multimodal data, which can weaken the fusion effect of multimodal features. To enhance the consistency among the sequence, atomic graph, and fragment graph representations, we employed the Normalized Temperature‐Scaled Cross Entropy Loss (NT‐Xent) to minimize the discrepancy across modalities. Specifically, for each molecule, its representations from different modalities are treated as positive pairs, while representations from other molecules are treated as negative pairs. This contrastive loss encourages the model to pull features of the same molecule closer and push apart those of different molecules, resulting in a more aligned and discriminative multimodal embedding space.

(14) $$L_{cl}=-\log\frac{\exp\left(\frac{\text{sim}\left(R_{S(I)},R_{A(I)}\right)}{T}\right)}{\sum_{J=1,I\neq J}^{N}\exp\left(\frac{\text{sim}\left(R_{S(I)},R_{A(J)}\right)}{T}\right)}$$

(15) $$-\log\frac{exp\left(\frac{sim\left(R_{A\left(I\right)},R_{F\left(I\right)}\right)}{T}\right)}{\sum_{J=1,I\neq J}^{N}exp\left(\frac{sim\left(R_{A\left(I\right)},R_{F\left(J\right)}\right)}{T}\right)}\;$$

(16) $$-\log\frac{exp\left(\frac{sim\left(R_{F\left(I\right)},R_{S\left(I\right)}\right)}{T}\right)}{\sum_{J=1,I\neq J}^{N}exp\left(\frac{sim\left(R_{F\left(I\right)},R_{S\left(J\right)}\right)}{T}\right)}$$

where *R*_*S*(*I*)_, *R*_*A*(*I*)_, and *R*_*F*(*I*)_ denote the sequence, atomic graph, and fragment graph representations of molecule *I*, respectively, while *R*_*S*(*J*)_, *R*_*A*(*J*)_, and *R*_*F*(*J*)_ denote the corresponding features of the molecule *J*. The function *sim*() denotes a similarity measure, and *T* is a temperature parameter that controls the sharpness of the similarity distribution.

In addition to the contrastive loss, we compute a prediction loss between the predicted output *P* and ground truth labels *Y*. Different loss functions are employed depending on the task type. For classification tasks, we employ BCEWithLogitsLoss, which combines sigmoid activation with binary cross‐entropy for stable training. For regression tasks, we employ the mean squared error (MSE) loss to quantify the discrepancy between predicted and true values. Since the contrastive loss and prediction loss differ in numerical scales, we introduce a balancing coefficient to regulate their relative contributions and ensure stable optimization. This multitask loss design jointly optimizes prediction accuracy and cross‐modal alignment, enhancing the robustness and interpretability of MF‐Net.

(17) $$L=l\left(P,Y\right)+\omega \cdot L_{cl}$$

where *l*() denotes the prediction loss function, *P* denotes the predicted labels, and *Y* denotes the truth labels.

### 4.9 Evaluation Metrics

To comprehensively evaluate the model's performance in the DTA prediction task, we employed four evaluation metrics: root mean square error (RMSE, the smaller the better), mean absolute error (MAE, the smaller the better), Pearson correlation coefficient (Rp, the larger the better), and standard deviation (SD, the smaller the better). RMSE quantifies the average deviation between the predicted and true values and is particularly sensitive to large errors, reflecting the overall bias of model predictions. MAE measures the mean of absolute prediction errors across all samples and provides a more robust estimate of prediction accuracy, especially in datasets with outliers. Rp evaluates the linear correlation between the predicted and observed values. SD measures the dispersion or volatility of data points within the dataset relative to the mean. For the structure‐based virtual screening task, we adopted the enrichment factor (EF, the larger the better) as the key evaluation metric, calculated at the top 0.1%, 0.5%, 1%, and 5% ranked subsets (denoted as EF0.1%, EF0.5%, EF1%, and EF5%). EF reflects the ability of the model to identify and enrich active compounds from a large chemical library, thereby indicating the effectiveness and accuracy of screening methods.

## Author Contributions

**Xiaojun Yao**: conceptualization, Writing – review and editing, methodology, project administration. **Yong Liang**: conceptualization, funding acquisition, writing – review and editing, project administration. **Xiang Zhang**: validation, writing – review and editing, methodology. **Shuo Liu**: conceptualization, data curation, investigation, methodology, validation, visualization, writing – original draft, writing – review and editing, funding acquisition. **Haixia Feng**: validation, writing – review and editing, methodology. **Xiaoqing Gong**: writing – review and editing, methodology. **Huanxiang Liu**: conceptualization, methodology, project administration, writing – review and editing, funding acquisition. **Yuquan Li**: writing – review and editing, methodology.

## Conflicts of Interest

The authors declare no conflict of interest.

## Supporting information




**Supporting File**: advs77345‐sup‐0001‐SuppMat.docx.

## Data Availability

All data sources used in this study are publicly available. The data and source code that support the findings of this study are openly available in MF‐Net at https://github.com/shuoliu0‐0/MF_Net/.
