# Supplementary material for: A Unified Hierarchical Multiscale Fusion Framework for Drug–Target Affinity Prediction: From Benchmark Performance to Nanomolar Inhibitor Discovery
Source: Adv Sci (Weinh). 2026 Aug 21:e77345. Online ahead of print. doi: 10.1002/advs.77345 (PMC13496275; doi:10.1002/advs.77345)
Supplement: Supplementary file 1 [file ADVS-9999-e77345-s001.docx]

**Supporting Information**

A Unified Hierarchical Multi-Scale Fusion Framework for Drug-Target Affinity Prediction: From Benchmark Performance to Nanomolar Inhibitor Discovery

Shuo Liu^1,3^, Xiang Zhang^1^, Haixia Feng^1^, Yuquan Li^4^, Xiaoqing Gong^2^, Yong Liang^1^*, Xiaojun Yao^2^*, Huanxiang Liu^2^*

^1^Chinese Medicine Guangdong Laboratory (Hengqin Laboratory), Guangdong-Macao In-Depth Cooperation Zone in Hengqin, 519000, China,

^2^Faculty of Applied Sciences, Macao Polytechnic University, Macao 999078, China,

^3^The Second Affiliated Hospital of Guangzhou University of Chinese Medicine, Guangzhou 510006, China,

^4^State Key Laboratory of Public Big Data, College of Computer Science and Technology, Guizhou University, Guiyang 550025, China,

^∗^Corresponding author. Tel: +853-8599-6874; Fax: +853-8599-6874; Email: Huanxiang Liu ([hxliu@mpu.edu.mo](mailto:hxliu@mpu.edu.mo)),Yong Liang ([yongliangresearch@gmail.com](mailto:yongliangresearch@gmail.com)), Xiaojun Yao ([xjyao@mpu.edu.mo](mailto:xjyao@mpu.edu.mo)).

Supplementary Figure S1. Distributions of protein sequence lengths and ligand SMILES lengths across the datasets used in this study. (a–c) Protein sequence length distributions in PDBBind v2016, DUD-E, and DEKOIS2.0, respectively. (d–f) Ligand SMILES length distributions in PDBBind v2016, DUD-E, and DEKOIS2.0, respectively.
